# Differential Responses of Hepatic Endoplasmic Reticulum Stress and Inflammation in Diet-Induced Obese Rats with High-Fat Diet Rich in Lard Oil or Soybean Oil

**DOI:** 10.1371/journal.pone.0078620

**Published:** 2013-11-06

**Authors:** Min Zhao, Baocai Zang, Mengjie Cheng, Yan Ma, Yanhong Yang, Nianhong Yang

**Affiliations:** 1 Department of Nutrition and Food Hygiene, Hubei Key Laboratory of Food Nutrition and Safety, Tongji Medical College, Huazhong University of Science and Technology, Wuhan, Hubei Province, People's Republic of China; 2 Ministry of Education Key Lab of Environment and Health, School of Public Health, Tongji Medical College, Huazhong University of Science and Technology, Wuhan, Hubei Province, People's Republic of China; Consiglio Nazionale delle Ricerche, Italy

## Abstract

**Scopes:**

To investigate the effects of high-fat diet enriched with lard oil or soybean oil on liver endoplasmic reticulum (ER) stress and inflammation markers in diet-induced obese (DIO) rats and estimate the influence of following low-fat diet feeding.

**Methods and Results:**

Male SD rats were fed with standard low-fat diet (LF, n = 10) and two isoenergentic high-fat diets enriched with lard (HL, n = 45) or soybean oil (HS, n = 45) respectively for 10 weeks. Then DIO rats from HL and HS were fed either high-fat diet continuously (HL/HL, HS/HS) or switched to low-fat diet (HL/LF, HS/LF) for another 8 weeks. Rats in control group were maintained with low-fat diet. Body fat, serum insulin level, HOMA-IR and ectopic lipid deposition in liver were increased in HL/HL and HS/HS compared to control, but increased to a greater extent in HL/HL compared to HS/HS. Markers of ER stress including PERK and CHOP protein expression and phosphorylation of eIF2α were significantly elevated in HL/HL group while phosphorylation of IRE1α and GRP78 protein expression were suppressed in both HL/HL and HS/HS. Besides, inflammatory signals (OPN, TLR2, TLR4 and TNF-α) expressions significantly increased in HL/HL compared to others. Switching to low-fat diet reduced liver fat deposition, HOMA-IR, mRNA expression of TLR4, TNF-α, PERK in both HL/LF and HS/LF, but only decreased protein expression of OPN, PERK and CHOP in HL/LF group. In addition, HL/LF and HS/LF exhibited decreased phosphorylation of eIF2α and increased phosphorylation of IRE1α and GRP78 protein expression when compared with HL/HL and HS/HS respectively.

**Conclusions:**

Lard oil was more deleterious in insulin resistance and hepatic steatosis via promoting ER stress and inflammation responses in DIO rats, which may be attributed to the enrichment of saturated fatty acid. Low-fat diet was confirmed to be useful in recovering from impaired insulin sensitivity and liver fat deposition in this study.

## Introduction

Over the decades, there has been a rapid increase in the prevalence of obesity worldwide [1], [2], [3]. Obesity is a known risk factor of many metabolic disease including atherosclerotic cardiovascular disease, type 2 diabetes and non-alcoholic fatty liver disease (NAFLD) [4], [5]. Collectively, these diseases constitute the greatest current threat to global public health. Therefore, both genetic and dietary obesity models have been used to study the mechanisms underlying the development and progression of obesity and related metabolic disorders intensively [6], [7], [8]. Chronic feeding of high-fat diets in either human or animals is known to provide a higher dietary energy density, to cause elevated serum free fatty acid (FFA), expanded adipose tissue and, subsequently obesity, which represented good models of obesity due to overconsumption of calories and fat in modern society[8], [9], [10], [11].

Increasing evidences demonstrated a chronically activated low-grade inflammation in diet-induced obesity, a phenomenon recently termed metabolically triggered inflammation (metaflammation) which primarily originated from adipose tissue and liver [12], [13], [14]. Being an important site of fatty acid oxidation, increased influx of lipid in the liver can undergo oxidative modification by lipoxygenases, cyclooxygenases, myeloperoxidase and generate oxidized phospholipids. Lipid metabolites including ceramide and diacylglycerol can induce insulin resistance and a series of proinflammatory molecules [15], [16], [17], [18]. Among the inflammatory molecules, osteopontin (OPN, *Spp1*) has been demonstrated to play a key role in hepatic inflammation disease via mediating the infiltration of neutrophils, macrophages and lymphocytes and inducing the expression of a variety of proinflammatory cytokines [19], [20]. Specially, toll like receptors (TLRs) were proposed to function as free fatty acid sensor which link insulin resistance and inflammation response and result in the production of proinflammatory cytokines such as tumor necrosis factor alpha (TNF-α) and interleukin 6 (IL-6) [21], [22]. Despite these findings, the precise mechanism underlying inflammation and obesity remains unknown.

Recent studies demonstrated that ER stress is a central feature of peripheral insulin resistance, lipogenesis, obesity and type 2 diabetes [23], [24]. Endoplasmic reticulum is an essential organelle for the folding and assembling of secretory and membrane proteins [25], [26]. When the ER homeostasis was interrupt by a series of stressors such as chemical toxicant, imbalance of ER calcium levels, glucose deprivation, lipid influx, and vital infection in vitro and vivo, the ER stress sensor glucose regulated protein (GRP78/Bip, *Hspa5*) triggered pathway termed unfolded protein response (UPR) was activated to cope with this stress[27], [28]. And several of these stress conditions occurred in high-fat diet-induced obesity (eg. lipid influx, glucose deprivation). The unfolded protein response functions via signaling through three branches, involving three type I transmembrane proteins: double-stranded RNA-activated protein kinase (PKR)-like ER kinase (PERK), inositol-requiring enzyme-1 (IRE-1) and activating transcription factor-6 (ATF6)[29], [30]. Recent studies have revealed that UPR plays a dual role in sustaining cellular homeostasis. When the stress was moderate, the UPR promotes degradation of improperly folded proteins and decreases the influx of protein to ER to protect the organism. But prolonged or unmitigated activity of the UPR would induce inflammation by activation of JNK and nuclear factor kappa B (NF-kB) pathways or cell death via CHOP (*Ddit3*) apoptosis signaling [29], [31], [32], [33]. IRE1α activated JNK has also been shown to be the key mediator of ER stress which disrupts insulin signal transduction through the phosphorylation of the insulin receptor substrate 1 (IRS-1) on serine307 [23], [34], [35]. In addition, both IRE1α -XBP1 and PERK-eIF2α pathway play a potential role in hepatic steatosis [36], [37].

Elevated ER stress was detected in adipose tissue and liver of both rodent obese model and obese patients [37], [38]. Recently, the concerns about the varied effects of different fat types on inflammation and metabolic abnormality were posed and confirmed by us and others [13], [39], [40], [41]. Acute effects of unsaturated and saturated fats on markers of ER stress and inflammation in rats have been compared utilizing intravenous infusion of soybean and lard oil. However, no study about the chronic effects of different dietary fat types on ER stress in diet-induced obese animal models has been reported. The present study aims to compare the effects of high-fat diet enriched with lard oil or soybean oil on hepatic ER stress and determine whether the effects can be reversed by a following 8-week low-fat diet feeding.

## Materials and Methods

### Ethics Statement

All animal experimental procedures were approved by the Animal Care and Use Committee of Huazhong University of Science and Technology (Permit number: S259). This study was conducted in strict accordance with the guidelines and authorization for the use of laboratory animals and we tried our best to minimize the suffering of affected animals.

### Animals, diets and experimental protocols

The animal model and the specific composition of diet have been described previously[13]. Briefly, 100 male Sprague-Dawley rats weighting 120–130 g were randomly assigned to 3 groups and fed with either low-fat standard chow (LF, n = 10) or two isoenergetic high-fat diets enriched with lard (HL, n = 45) or soybean oil (HS, n = 45) respectively. Dietary intake was recorded daily and body weight was measured weekly in the morning throughout the study. After 10 weeks of free access to their corresponding diets, rats in HL or HS group with body weights more than 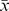
+1.96 s of LF group were classified as diet-induced obesity (DIO) and selected for further study. For the additional 8 weeks, the LF remained on their original diet and the DIO rats from HL and HS group were randomly subdivided into two groups respectively. One subgroup of DIO rats were kept on their original high-fat diets (HL/HL and HS/HS) and the others were switched to low-fat standard chow (HL/LF and HS/LF). At the end of the experiment, all rats were sacrificed after 12 h fasting and trunk blood was collected and serum was stored at −80°C for further use. Liver and white adipose tissue from both perirenal and epididymal locations were dissected, weighed and snap-frozen in liquid nitrogen immediately and stored at −80°C for further use. A piece of each liver was fixed with formaldehyde for histology. Body fat percentage was calculated as [(perirenal fat+epididymal fat)/final body weight]×100%.

### Serum measurements

Blood glucose concentrations were determined by the glucose oxidase method with a commercial kit (Jiancheng Bioengineering Institute, Nanjing, China). Total cholesterol (TC) and triglyceride (TG) were determined by enzymatic colorimetric assays using commercial enzyme kits (Biosino Bio-Technology & Science Inc, Beijing, China), and serum insulin concentrations were measured by radioimmunoassay (Chemclin Biotechnology Corporation Limited, Beijing, China). Homeostasis model assessment of insulin resistance (HOMA-IR) was calculated according to the following formula: HOMA-IR =  fasting insulin (μU/mL)×fasting glucose (mmol/L)/22.5.

### Liver triglyceride content assay

Frozen liver segments (100 mg) were homogenized in isopropanol with the proportion of 1∶9 (weight/volume), then centrifuged (5,000 rpm, 15 min) and the supernatants were used for triglyceride (TG) determination using kit by GPO-PAP method (Biosino Bio-Technology & Science Inc, Beijing, China).

### Liver histology

Formaldehyde fixed liver tissues were embedded in paraffin. Five-micrometer-thin sections were obtained and stained with hematoxylin and eosin (HE). Liver sections were observed and photographed by fluorescence microscope (Nikon, Japan). Semi-quantitative estimations of liver fat vacuoles were archived by using Image Pro-Plus 6 (IPP).

### Analysis of gene expressions

Total RNA was extracted from frozen liver tissue samples using TRIZOL (Invitrogen, USA) according to the manufacturer's protocol. 3.0 µg of the total RNA was reverse-transcribed by Revert Aid First Strand cDNA synthesis kit (Fermentas, CA). Real-time quantitative PCR was performed using the SYBR Premix Ex Taq™ (TaKaRa Bio Inc.) according to the manufacturer's instructions on an ABI 7900HT real-time PCR system (Applied Biosystems, Foster, CA, USA) with primers as shown in table 1. Standard curves for each primer pair were generated by serial dilutions of cDNA from a reference sample and used for regression analyses. All PCR assays were performed in triplicate. The variance of the triplicate measurements was <1%. Results were analyzed using the standard curve method by the SDS (Sequence Detection Systems) software. The data was expressed as the relative levels of mRNA after normalized with GAPDH.

**Table 1:** Primer sequences for PCR analysis.

mRNA	Primer		Product size (bp)
Tlr4	Forward	5′-GCAGAAAATGCCAGGATGATG -3′	110
	Reverse	5′-AAGTACCTCTATGCAGGGATTCAAG -3′	
Tlr2	Forward	5′-GCTGTTGCGTTACATCTTG-3′	133
	Reverse	5′-CCGTATTGTTACCGTTTCTAC-3′	
OPN (*Spp1*)	Forward	5′-ATAGCTTGGCTTACGGACTGA-3′	142
	Reverse	5′-GCAACTGGGATGACCTTGATAG-3′	
Chop (*Ddit3*)	Forward	5′-CCTTCACTACTCTTGACCCTG-3′	174
	Reverse	5′-GACCACTCTGTTTCCGTTTC-3′	
GRP78(*Hspa5*)	Forward	5′-GATAATCAGCCCACCGTAA-3′	192
	Reverse	5′-TCCTGTCCCTTTGTCTTCA-3′	
TNF-α	Forward	5′-GGAAAGCATCCGAGATG-3′	145
	Reverse	5′-CAGTAGACAGAAGAGCGTGGT-3′	
PERK	Forward	5′-CTCCTGTCTTGGTTGGGTCTG-3′	218
	Reverse	5′-CTTCTTGCGGATGTTCTTGCT-3′	
ATF6	Forward	5′-GGATTTGATGCCTTGGGAGTCAGAC-3′	163
	Reverse	5′-ATTTTTTTCTTTGGAGTCAGTCCAT-3′	
XBP1	Forward	5′-GAATGCCCTGGTTACTGAAGAG-3′	205
	Reverse	5′-CCAAAAGGATATCAGACTCAGAATC-3′	
XBP1s	Forward	5′-GAGTCCGCAGCAGGTG-3′	65
	Reverse	5′-GCGTCAGAATCCATGGGA -3′	
SREBP-1c	Forward	5′- CGCTACCGTTCCTCTATCA-3′	165
	Reverse	5′- TCGCAGGGTCAGGTTCT-3′	
GAPDH	Forward	5′-GCAAGTTCAACGGCACAG-3′	140
	Reverse	5′-GCCAGTAGACTCCACGACAT-3′	

### Protein isolation and Western blotting

Liver tissues were homogenized in ice-cold buffer containing 50 mM Tris, pH 7.5, 150 mM NaCl, 1% Nonidet P-40, 1% sodium deoxycholate, 1% sodium dodecyl sulfate, 0.1 mM dithiothreitol, 0.05 mM phenylmethyl sulfonylfuoride, 10 mM NaF, 0.5 mM Na3Vo4, protease inhibitor cocktail (Amresco, Solon, OH) and phosphatase inhibitor cocktail (Thermo Scientific, US) and centrifuged at 12 000 g for 15 min at 4°C. The supernatant was collected and protein content was determined by a protein assay reagent from Bio-Rad (Hercules, CA). A sample of 60 µg protein were mixed with sodium dodecyl sulfate sample buffer and boiled for 5 minutes, separated by sodium dodecyl sulfate polyacrylamide gel electrophoresis on 8% or 10% polyacrylamide gels, transferred to polyvinylidene difluoride membranes and blocked 1.5 hours with 5% milk in TBS-0.05% Tween. The membranes were then incubated with the primary antibody OPN (sc-21742; Santa Cruz), CHOP (L63F7; Cell Signaling), total and phosphorylated PERK (sc-20790; Santa Cruz and #3179; Cell Signaling), total and phosphorylated IRE1α (sc-20790; Santa Cruz and ab48187; abcam), GRP78 (#3183; Cell Signaling), total and phosphorylated eIF2α (#5324 and #3398; Cell Signaling) and β-actin (8H10D10; Cell Signaling) overnight at 4°C. After washing with TBS-T for 3 times, membranes were incubated for 1 h at ambient temperature with horseradish peroxidase-conjugated antibodies (Pierce). Signals were detected using a commercial chemiluminescence enhancement kit (Super Signal West Pico; Pierce).

### Statistical analysis

Statistical analyses were conducted using SPSS software (version 13.0; SPSS Inc, Chicago, IL). Comparisons of the data between different groups were performed by one-way ANOVA, and SNK-q was used for comparison between 2 groups. Data were expressed as mean ± SEM and *P*<0.05 were considered statistical significant.

## Results

### Body weight, body fat accumulation and HOMA-IR were increased in HL/HL and HS/HS and decreased when switched to low-fat diet

**Figure 1:**
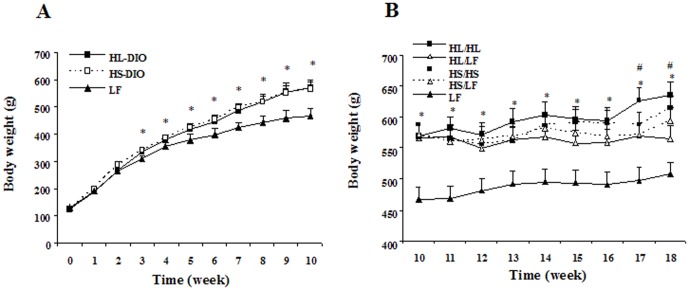
Differential effects of high-fat diets rich in lard or soybean oil on body weight of male SD rats. Body weights were measured weekly. **(A)** Body weight of each group in the first 10 weeks. **(B)** Body weight of each group in the following 8 weeks. Data were presented as mean ± SEM. ^*^ *P*<0.05 for all the four groups VS. LF, ^#^ *P*<0.05 for HL/HL VS. HL/LF

Obese rats had significantly increased body weight compared to LF rats starting from the 3rd week (Fig. 1A). In the following 8 weeks, rats of HL/LF and HS/LF groups showed significantly reduced weight gain compared with rats with continued high-fat diets, while no statistical difference was found between HL/HL and HS/HS group (Fig. 1B). DIO rats of HL/HL or HS/HS had higher intra-abdominal fat, body fat percentage, serum insulin and HOMA-IR compared with LF group (*P*<0.05) as presented in table 2. Meanwhile, as compared to HL/HL, all the parameters mentioned above were significantly lower in HS/HS group although no difference in initial body weight, weight gain and energy intake throughout the experiments was observed. Both HL/LF and HS/LF showed significant decrease in intra-abdominal fat, body fat percentage, serum insulin and HOMA-IR when compared to their respective counterparts on continuous high-fat diet. Serum glucose was comparable among all five groups and TC levels were higher in both HL/HL and HS/HS group than in LF, while TG was only higher in HL/HL group.

**Table 2:** Body fat and serum parameters.

	HL/HL	HL/LF	HS/HS	HS/LF	LF
Number of rats	8	8	8	8	10
Perirenal+epididymalfat(g)	43.74±4.79a,c	24.07±3.58a,b	29.98±7.45^a^	22.14±5.28^b^	18.04±6.00
Body fat percentage (%)	6.89±0.70^ac^	4.05±0.48^ab^	4.57±0.77^a^	3.65±0.58^bc^	3.10±0.73
TC(mmol/L)	1.09±0.26^a^	0.83±0.15	1.13±0.25^a^	0.86±0.28	0.79±0.21
TG(mmol/L)	0.76±0.25^a^	0.55±0.15	0.55±0.23	0.38±0.15	0.43±0.09
GLU(mmol/L)	5.32±0.22	4.13±0.53	5.28±0.40	4.89±0.41	5.13±0.55
Insulin(uIU/ml)	33.47±10.79^ac^	14.02±3.92^b^	21.45±6.95^a^	12.48±5.04^b^	15.29±2.19
HOMA-IR	7.88±2.34^ac^	2.71±1.02^b^	5.29±2.17^a^	2.64±0.94^b^	3.42±0.62

Data are shown as mean ± SD;

a *P*<0.05 compared with the LF group;

b *P*<0.05 compared with corresponding group received respective continuous high-fat diet;

c *P*<0.05 compared with corresponding group received HS.

### Liver fat deposit was increased in HL/HL and HS/HS compared to LF with greater extent in HL/HL, and decreased in HF/LF and HS/LF by low -fat diet feeding

**Figure 2:**
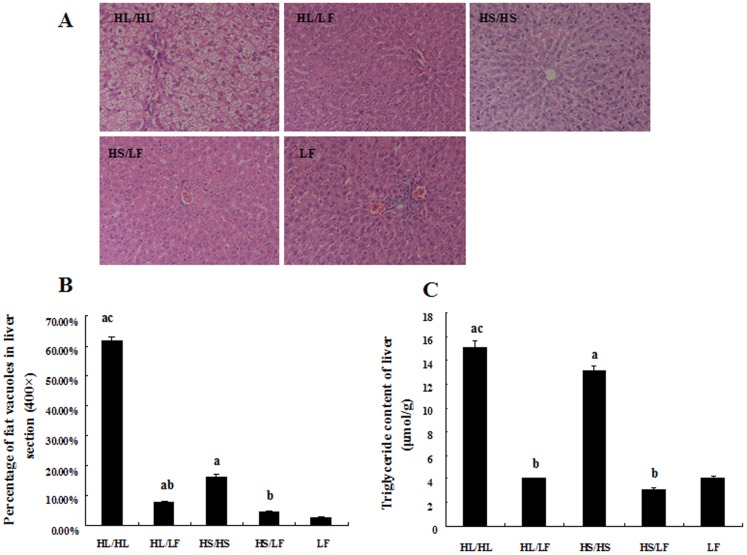
Liver fat deposition in each group assayed at 18^th^ week. **(A)** HE staining of liver tissue was used to assess lipid accumulation. **(B)** Semi-quantitative assessment of liver lipid droplets were presented as percentage of fat vacuoles in liver section by using image-pro plus 6 (400×), N = 5. (C) Liver TG content was detected by GPO-PAP method in liver tissue homogenate. Data were shown as Mean ± SEM, N = 5. ^a^ *P*<0.05 versus LF group, ^b^ *P*<0.05 versus corresponding group received respective continuous high-fat diet, ^c^ *P*<0.05 versus corresponding group received HS.

Liver fat accumulation was assessed by histological analysis of liver sections and triglyceride measurement of liver tissue homogenate. DIO rats in HL/HL group had the highest percentage area occupied by fat vacuoles in the sections under microscope (Fig. 2A, 2B) and highest liver TG content (Fig. 2C). Liver TG contents and fat vacuoles also increased, but to a lesser extent in HS/HS group. Switching to low-fat diet dramatically decreased the liver TG content and lipid droplets of DIO rats in HL/LF and HS/LF groups.

### Hepatic inflammation response was activated in HL/HL and HS/HS groups and alleviated in HL/LF and HS/LF groups

**Figure 3:**
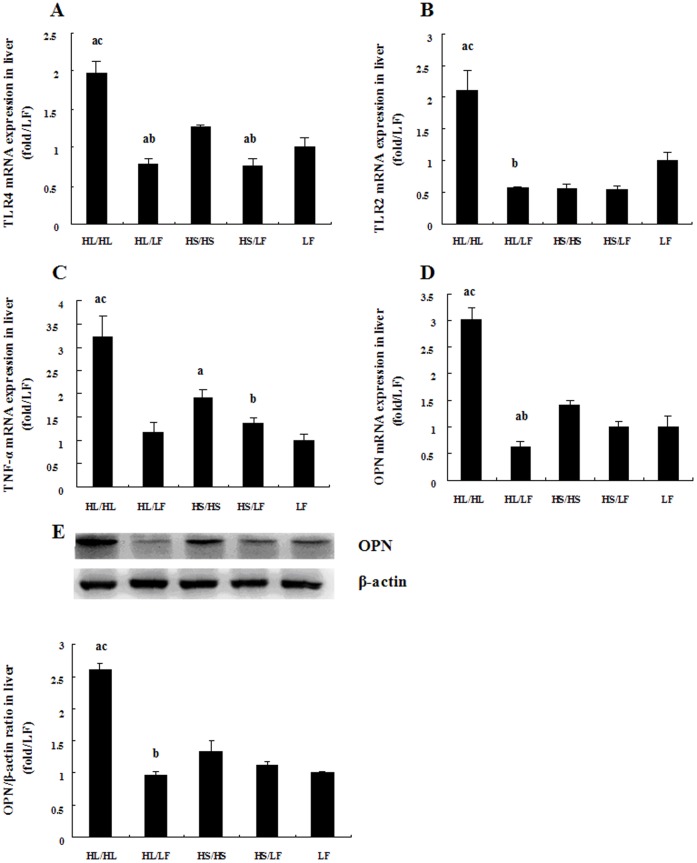
Comparative effects of different diets on inflammatory molecules expressions. Relative TLR4 (A), TLR2 (B), TNF-α (C) and OPN (D) mRNA expressions in liver tissue, N = 5. (E) Representative Western blot analysis of OPN and β-actin proteins in liver tissue of rats. Bar graph represents quantification of OPN levels normalized by β-actin levels, N = 5. Data was presented as Mean ± SEM. ^a^ *P*<0.05 versus LF group, ^b^ *P*<0.05 versus corresponding group received respective continuous high-fat diet, ^c^ *P*<0.05 versus corresponding group received HS.

Hepatic inflammation response was dramatically activated in HL/HL group featured by highest levels of OPN protein and OPN, TLR4, TLR2, TNF-α mRNA expressions among all groups (Fig. 3). The HS/HS group exhibited higher expression of TNF-α mRNA but no difference in TLR4, TLR2 and OPN mRNA as well as OPN protein when compared with LF group. Low-fat diet significantly decreased the levels of TLR2, TLR4, OPN, TNF-α mRNA and OPN protein in HL/LF group and TLR4, TNF-α mRNA in HS/LF group as compared with their respective counterparts continuing on high-fat diets.

### Increased PERK protein and phosphorylation of eIF2α were induced by both lard and soybean oil and reversed by following low-fat feeding

**Figure 4:**
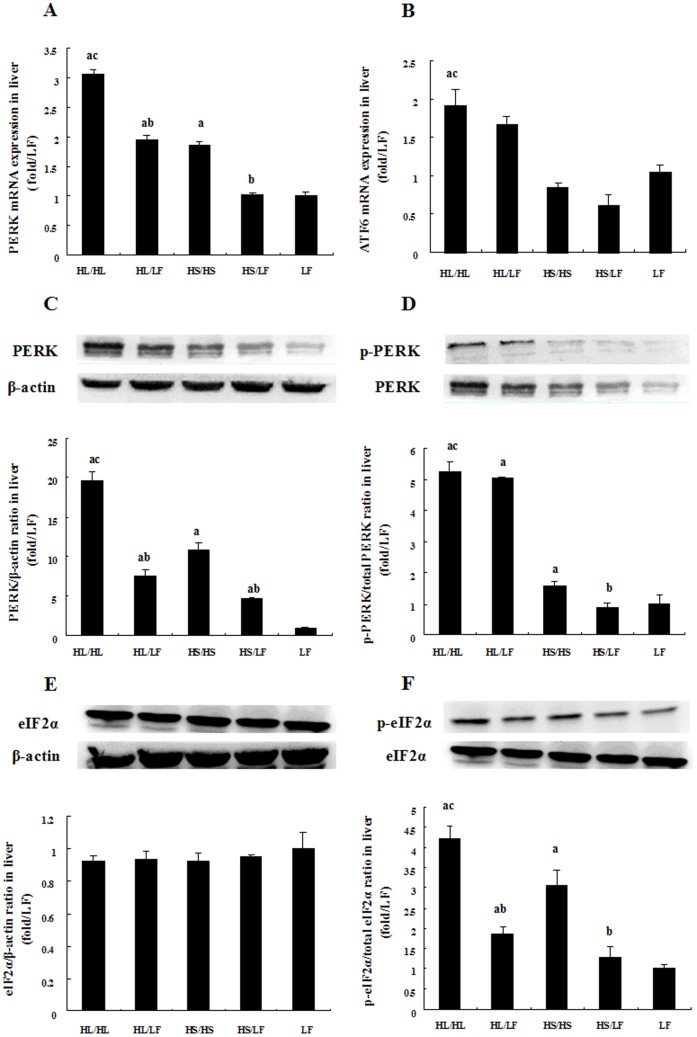
Comparative effects of different diets on PERK/eIF2α pathway activation and ATF6 mRNA expression in liver tissues. Relative PERK (A) and ATF6 (B) mRNA expression among groups, N = 5. Representative western blot analysis of total and phosphorylated PERK and eIF2α proteins in liver tissues of rats. Bar graph represents quantification of PERK (C) and eIF2α (E) protein expression normalized by β-actin, and phosphorylation of PERK (D) and eIF2α (F) levels normalized total PERK or eIF2α levels respectively, N = 5. Data was presented as Mean ± SEM. ^a^ *P*<0.05 versus LF group, ^b^ *P*<0.05 versus corresponding group received respective continuous high-fat diet, ^c^ *P*<0.05 versus corresponding group received HS.

PERK is one of the three most ER-proximal regulators which is activated through autophosphorylation and phosphorylates alpha-subunit of eukaryotic translation initiation factor (eIF2α), leading to attenuation of general protein synthesis [42]. Fig. 4 demonstrated that the levels of PERK mRNA, total and phospho-PERK protein and phospho-eIF2α expressions were upregulated in HL/HL and HS/HS groups when compared to LF, with greater extent in HL/HL group. Switching to low-fat diet significantly attenuated the increases in HL/LF and HS/LF compared to their respective high-fat diet counterparts. In addition, as shown in Fig. 4B, ATF6 mRNA was increased in HL/HL compared with HS/HS and LF group.

### IRE1α/XBP1 pathway was suppressed in HL/HL and HS/HS and the suppression was attenuated by following low-fat feeding

**Figure 5:**
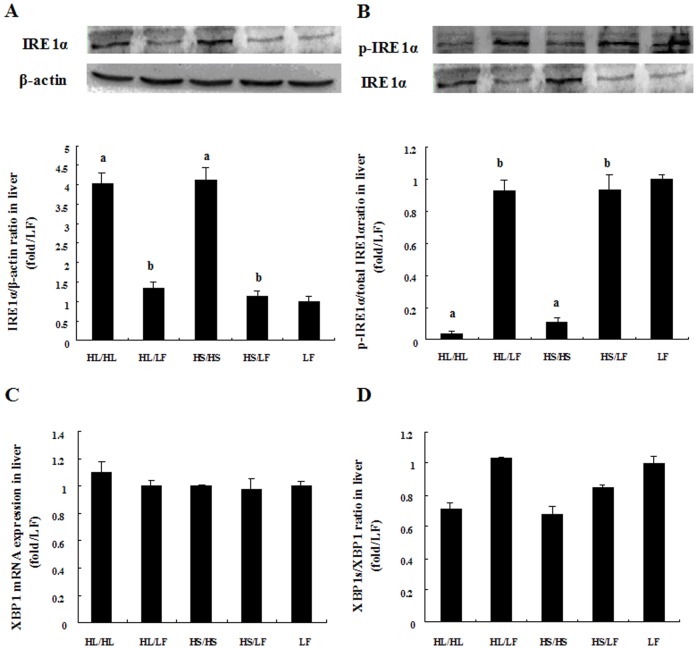
Comparative effects of different diets on IRE1α/XBP1 pathway activation in liver tissues. (A) Representative western blot analysis of Total and phosphorylated IRE1α proteins in liver tissue of rats. Bar graph represents quantification of IRE1α protein expression levels normalized by β-actin levels and phosphorylation of IRE1α normalized by t IRE1α level, N = 5. **(B)** Relative XBP1 mRNA expression and ratio of spliced XBP1 among groups, N = 5. Data was presented as Mean ± SEM. ^a^ *P*<0.05 versus LF group, ^b^ *P*<0.05 versus corresponding group received respective continuous high-fat diet, ^c^ *P*<0.05 versus corresponding group received HS.

IRE1 represents the most conventional pathway of UPR which undergoes aggregation and autophosphorylation upon activation. Activated IRE1α functions as endoribonuclease which specifically splices the X-box-binding protein 1 (XBP1) mRNA resulting in the coding of a transcription factor spliced-XBP1 (XBP1s), which induces UPR target genes [43]. As demonstrated in Fig. 5A and 5B, the levels of total IRE1α protein was increased but phosphorylation of IRE1α was decreased in HL/HL and HS/HS compared to LF group, and these changes were attenuated in HL/LF and HS/LF by low-fat diet feeding. Although similar expression of XBP1 mRNA expressions were detected among groups, spliced XBP1 were lower in HL/HL and HS/HS but with no statistical significance (Fig. 5C, 5D).

### ER chaperone GRP78 was reduced in HL/HL and pro-apoptosis transcription factor CHOP was elevated in HL/HL

**Figure 6:**
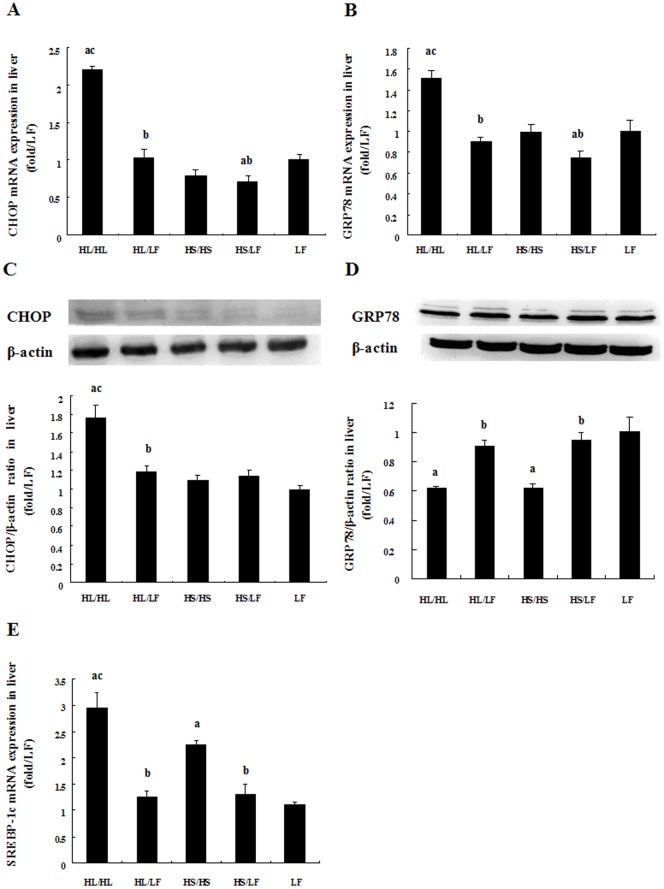
Comparative effects of different diets on GRP78, CHOP, SREBP-1c and ATF6 expressions in liver tissues. Relative GRP78 (A), CHOP (B) and SREBP (E) mRNA expression in liver. Representative western blot analysis of GRP78 (C) and CHOP (D) proteins in liver tissue of rats. Bar graph represents quantification of protein expression levels normalized by β-actin levels, N = 5. Data was presented as Mean ± SEM. ^a^ *P*<0.05 versus LF group, ^b^ *P*<0.05 versus corresponding group received respective continuous high-fat diet, ^c^ *P*<0.05 versus corresponding group received HS.

Both GRP78 and CHOP act as apoptotic regulator by protecting or promoting cell death during ER stress [28], [44]. They are good measurements of ER stress. As shown in Fig. 6, both CHOP mRNA and protein expressions were highest among all groups while switching to low-fat diet significantly decreased their expressions in HL/LF. CHOP mRNA expression in HS/LF was also significantly decreased compared to HS/HS. Similarly, GRP78 mRNA was upregulated in HL/HL group compared to LF and HS/HS and significantly decreased in HL/LF and HS/LF group when switched to low-fat diet. Despite the upregulation of GRP78 mRNA in HL/HL, its protein expression was significantly reduced while switching to low-fat diet reversed the changes. The levels of SREBP-1c mRNA expression were upregulated in HL/HL and HS/HS, and were reversed by low-fat diet feeding in HL/LF and HS/LF.

## Discussion

The present work showed that DIO rats featured increased body weight compared with the control group, in accordance with previous studies reported by us and others[13], [45], [46]. In parallel with increased body weight, body fat mass, serum lipid profiles, insulin and hepatic steatosis were elevated in both HL/HL and HS/HS at the end of experiment, but were increased to a greater extent in HL/HL group. The results suggested that high-fat diet enriched with lard oil or soybean oil had similar ability of inducing obesity but different extent of effects on fat deposition, hepatic inflammation and ER stress. Withdrawal of the dietary fat in the following 8 weeks of feeding reversed the deleterious effect of both kinds of high-fat diets. This may be explained by less saturated fatty acid and higher monounsaturated fatty acid and polyunsaturated fatty acid in soybean oil than lard oil. Besides, there is no cholesterol and much more vitamin E as well as isoflavone in soybean oil than in lard oil which had been demonstrated in previous studies with beneficial effect of lessening insulin resistance and oxidative damage[47], [48].

Inflammation response was demonstrated as an important mechanism which leads to and promotes insulin resistance and hepatic steatosis in previous studies [12], [49]. Obesity is associated with chronic low grade inflammation, characterized by insulin resistance, often accompanied by hepatic steatosis [14], [50], [51]. High-fat diets, especially high-fat diet enriched with saturated fatty acid exert more deleterious effect on insulin sensitivity and inflammation response [13], [52], [53]. The possible mechanism involved activation of TLRs and consequent induction of TNF-α [21], [54]. In line with previous reports, our results showed that rats kept on diet enriched with lard oil exhibited higher TLR4, TLR2, TNF-α mRNA expressions when compared to rats fed with standard chow or diet enriched with soybean oil [21], [49], [55]. Osteopontin (OPN) is an extracellular matrix protein and inflammatory cytokine which has recently been shown to be involved in human and murine obesity and hepatic steatosis[20]. Agonists of TLR4 has been shown to upregulate OPN expression, while antagonists of which were reported to lead to down regulation of OPN expression [56], [57]. OPN knockout mice and mice interfered with anti-OPN antibody were protected from peripheral insulin resistance compared to their wild-type controls when fed with high-fat diet [19], [58], [59]. In the present study, significantly elevated OPN expression in HL/HL group compared with HS/HS and LF group were consistent with increased TLRs and liver fat deposit, and switching to low-fat diet resulted in reversion of OPN and TLRs expressions, along with hepatic steatosis, which further proved the effect of dietary fat rich in SFA on OPN expression by TLRs and the involvement of OPN in insulin resistance and hepatic steatosis.

Recent studies have highlighted ER stress as a link between insulin resistance, inflammation and hepatic steatosis [23], [24], [31]. The primary function of UPR is to reestablish ER homeostasis by degrading misfolded proteins and reducing protein synthesize. It would also induce apoptosis, lead to immune response and promote hepatic steatosis if the stress cannot be resolved. All the three main branches of UPR can trigger inflammatory response through activation of NFκB pathway while IRE1 pathway can also activate the JNK pathway through TRAF2 (TNF receptor associated factor 2)[21], [60] and regulate hepatic lipogenesis through activation of genes such as SREBP1c and GADD34 [23], [24], [31]. Studies demonstrated that TLR2 and TLR4 activated IRE1α/XBP1 pathway was required for optimal and sustained production of proinflammatory cytokines in macrophages [60]. In the present study, consistent with severer insulin resistance and hepatic lipid infiltration, elevated SREBP-1c mRNA and PERK, phospho-eIF2α and CHOP protein expressions concomitant with decreased GRP78 protein expressions were significantly induced by continuous feeding of high-fat diet enriched with lard oil in HL/HL group. In accordance with previous studies aimed to compare the effect of different fat types on ER stress in vitro and in vivo, we proposed that SFA can induce severer ER stress than PUFA [61], [62], [63].

However, in the present work, activated IRE1α protein expression was significantly suppressed in HL/HL and HS/HS than LF group, and the suppression was reversed by withdrawal of the enriched fat in the following low-fat feeding. This is in discrepancy with several previous reports [64], [65] which showed increased activation of IRE1α. Lin et al [66] demonstrated that IRE1 pathway was firstly inactivated, followed by attenuated ATF6 activities several hours later during persistent ER stress in human cells while PERK signaling, including translational inhibition and proapoptotic transcription regulator CHOP induction, was maintained. Their findings suggested that varied time courses of the individual UPR branches influence the cell's ultimate fate in response to ER stress. In a recent study by Zhang et al [67], after treatment with an ER stress-inducer, hepatocyte-specific IRE1α deletion mice developed severe hepatic steatosis compared with wild type mice. The two studies above and our own work suggested a protective role of IRE1α/XBP1 pathway which distinguishes from PERK/CHOP pathway in metabolic disorders. On the basis of these studies, we can speculate that IRE1α protein was induced with the onset of stress but experienced dephosphorylation during prolonged ER stress. The dephosphorylation of IRE1α had been previously demonstrated in prolonged glucose stimulated pancreatic β cells and tunicamycin stimulated HEK293 cells respectively [68], [69]. Similarly, longer time and persistent high-fat feeding in HL/HL and HS/HS might be a possible explanation for the increased total IRE1α but decreased phospho-IRE1α protein observed in this study. And the increased GRP78 mRNA in HL/HL was presumably due to still activated ATF6 pathway at that time, which also targets GRP78 transcriptionally [70] while the possible explanation of decreased GRP78 protein in HL/HL and HS/HS included reduction in translation and rapid degradation of GRP78 protein, although further studies are required to address this process in detail. Also, further studies to investigate the underlying mechanism for different response of PERK/CHOP and IRE1α/XBP1 pathway to long term high-fat diet would help understanding the pathogenesis of diet-induced obesity.

## Conclusion

In summary, the present study suggested lard oil rich in SFA can promote severer ER stress, inflammation, hepatic steatosis and insulin resistance than soybean oil rich in polyunsaturated fatty acid. The modest ER stress and inflammation in HS/HS group may be attributing to the antagonistic effect of polyunsaturated fatty acid to the amplification of UPR [61] but we could not exclude the contribution of other bioactive compounds such as isoflavone and Vitamin E contained in soybean oil. And, to the best of our knowledge, this is the first study to compare the ER stress response to high-fat diet enriched with lard oil and soybean oil and indentify the validity of low-fat diet intervention in alleviating the ER stress in high-fat diet induced obese rats. Despite abundant evidences emerged demonstrating the important role of ER stress in obesity-related insulin resistance and NAFLD, studies are warranted to further clarify the causal relationship among ER stress signaling pathways, the origin of obesity, insulin resistance and NAFLD.
